# Clinical and Economic Evaluation of Transdermal Oxybutynin in the Treatment of Overactive Bladder in Spain

**DOI:** 10.36469/9811

**Published:** 2018-01-09

**Authors:** Carlos Crespo, Pedro Blasco, Marcelo Guigini, Jordi Galván

**Affiliations:** 1 GM Department of Statistics Universita de Barcelona, Barcelona, Spain; Axentiva Solutions, Sta. Cruz de Tenerife, Spain; 2 Department of Urology Hospital Universitario de Valme, Seville, Spain; 3 Medical and Regulatory Affairs Department Laboratorios Gebro Pharma S.A., Barcelona , Spain

**Keywords:** oral muscarinic antagonists, mirabegron, persistence, cost–effectiveness, oxybutynin transdermal patch, idiopathic overactive bladder

## Abstract

**Background:** This study evaluated the clinical outcomes and economic results of transdermal oxybutynin compared to fesoterodine, tolterodine, solifenacin, oxybutynin, trospium chloride, and mirabegron for overactive bladder syndrome in Spain.

**Materials and Methods:** A Markov model was built with monthly cycles for a 5-year time frame. The model reflected clinical events, discontinuation, dose scaling and change in treatment according to actual clinical practice. Based on experts’ opinion and the literature, the use of resources and Spanish costs were incorporated into the model. The measure of efficiency used was the cost per quality-adjusted life year (QALY) gained. The economic evaluation was performed from the perspective of the Spanish healthcare system, discounting costs (€2017) and effects at 3%. The robustness of the results was validated with a deterministic and probabilistic sensitivity analysis.

**Results:** After a year, transdermal oxybutynin was seen to have greater persistence deriving from its better risk/benefit balance compared to muscarinic antagonists and mirabegron (55% transdermal oxybutynin, 33% mirabegron, 25% tolterodine, 27% fesoterodine, 25% solifenacin, 23% trospium chloride and 17% oxybutynin). At 5-years, better persistence resulted in improvements in QALY gained by transdermal oxybutynin of 0.050, 0.040, 0.039, 0.038, 0.034 and 0.010 compared to oxybutynin, fesoterodine, solifenacin, trospium chloride, tolterodine and mirabegron, respectively. The incremental cost–effectiveness ratio of transdermal oxybutynin ranged from €1313.96 per QALY gained compared to fesoterodine to €14 101.57 per QALY gained compared to trospium chloride.

**Conclusions:** Kentera® (transdermal oxybutynin) is a cost–effective treatment in overactive bladder syndrome compared to muscarinic antagonists and mirabegron.

## BACKGROUND

### Disease Burden

Overactive bladder (OAB) syndrome is defined by the presence of urinary urgency, with or without urge urinary incontinence (UUI), often accompanied by urinary frequency and nocturia.[1] It is estimated that there are approximately 1.5 million people with OAB in Spain[2]; however, only 16.7% of patients with OAB receive treatment.[3]

OAB has a significant negative impact on the quality of life (QoL) of patients, their family members and their caregivers, since it affects social function, sexual function, interpersonal relationships and work life.[1,4] In addition, UUI has been associated with an increase in the use of resources and a decrease in the productive capacity of working-age patients.[5,6]

### Therapeutic Lines for OAB

Treatment options for OAB range from conservative management (lifestyle recommendations, bladder training, etc.) to drug treatment and surgery where needed.[1] Drug treatments include muscarinic antagonists and beta-3 adrenergic agonists with a grade of recommendation level of A. Muscarinic antagonists are associated with adverse effects, including dry mouth, constipation and dry eye. In clinical practice, this is among the reasons why patients end up discontinuing treatment.[7,8] However, transdermal oxybutynin (OXY-TDS) (Kentera®) shows a similar efficacy to all other muscarinic antagonists but with a better safety profile.[9-11] These characteristics are unique to OXY-TDS and promote greater treatment persistence, and therefore greater continence, among patients.[12]

A recent meta-analysis that included 44 randomized clinical studies with 27 309 patients showed mirabegron (MI) to be as effective as muscarinic antagonists in reducing UUI and uriary frequency (except solifenacin [SO] 10 mg, which showed greater efficacy).[13] However, this study excluded OXY-TDS without further explanation,[9-11] although the bibliographical reference was included in the review. Due to the OXY-TDS persistence[12] and the importance in the Spanish market,[14] we believe in the need of a comparison with all pharmacological OAB treatments. Thus, we felt compelled to evaluate the cost-effectiveness of OXY-TDS to all other muscarinic antagonists and MI for idiopathic OAB in Spain.

## MATERIALS AND METHODS

A Markov model was built with monthly cycles to reflect the natural history of patients with idiopathic OAB after failure of conservative treatments in Spain for 5 years.[15] The cycle length was selected to capture the discontinuation of OAB patients. The treatments evaluated were as follows:

- Transdermal oxybutynin (OXY-TDS)
- Fesoterodine (FE)
- Tolterodine (TO)
- Solifenacin (SO)
- Oxybutynin (OX)
- Trospium chloride (TR)
- Mirabegron (MI)

The measures of effectiveness analyzed were months of initial treatment persistence, proportion of patients who discontinued and quality-adjusted life years (QALYs). To estimate the proportion of patients who discontinued, the number of patients who discontinued treatment was calculated monthly for 5 years. The results of the cost–effectiveness analysis were expressed using the incremental cost–effectiveness ratio (ICER) for OXY-TDS versus muscarinic antagonists and MI, calculated using the following formula:

$$ICER = \frac{Cost_{OXY-TDS} - Cost_{alternative}}{QALYs_{OXY-TDS} - QALYs_{alternative}}$$

where QALYs are quality-adjusted life years.

OXY-TDS was identified as cost–effective when it was less costly and more effective than the alternative and when the ICER compared to the alternative was below the threshold of willingness to pay per QALY gained assumed in Spain (between €25 000/QALY gained[16] and €30 000/QALY gained[17]).

The study was conducted from the perspective of the Spanish National Health System; therefore, it included only direct medical costs. As recommended by the main Spanish economic evaluation guideline, a discount rate of 3% was applied, both for costs (€2017) and for effects.[18] Model was built in Excel.

**Rationale of the Model**

At the start of the model, patients received one of the treatments evaluated according to the dosing regimen recommended in the corresponding summaries of product characteristics based on Spanish Guidelines.[1,19] It was assumed that all patients from the start were continent during the first month. At the end of each cycle, disease progression was evaluated according to patients' continence or incontinence and treatment persistence (Figure 1). At any time, patients could move from one state of health to another and could suffer from adverse effects related to their prescribed treatment. Only adverse effects (dry mouth and constipation) resulting in a decrease in quality of life or incurring costs were included in the study. Mortality was not taken into account as there were no differences in terms of mortality between treatments. The model ended 5 years after it started.

**Figure 1.** Pharmacoeconomic Model Structure

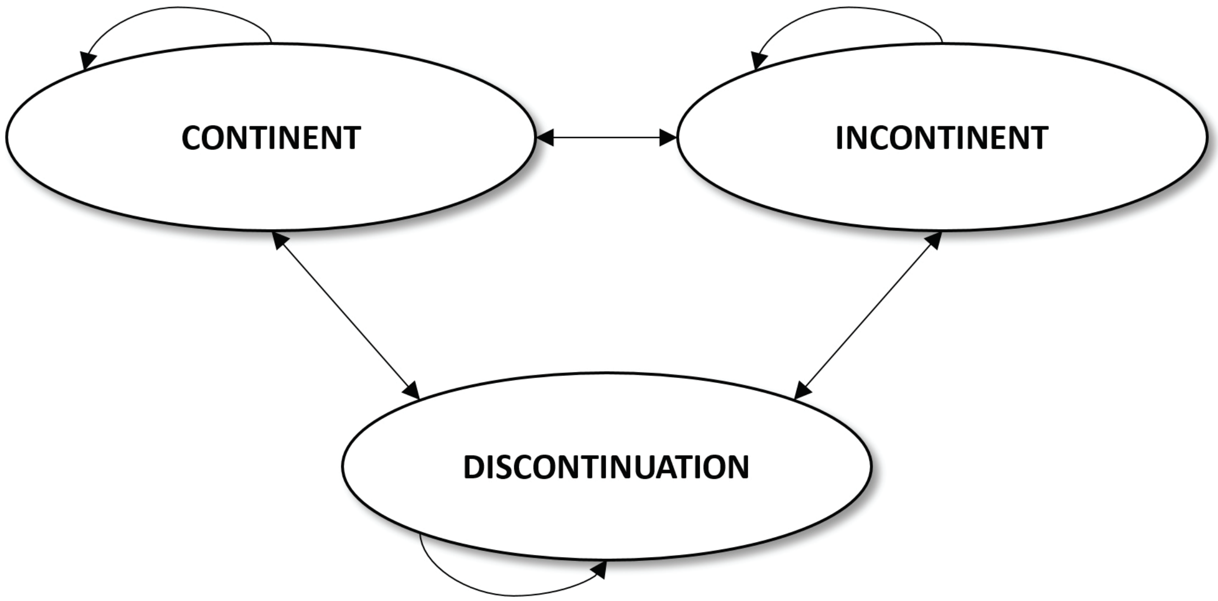

Patients were refractory to drug treatment when they failed to respond to two consecutive oral medications before being prescribed Botox or neuromodulation. Patients with FE and SO started with the low dose and had their dose scaled up before changing treatment. At any time, patients could discontinue or restart the same treatment or a different one (Figure 2). When the muscarinic antagonist and MI treatment lines had failed, based on the expert panel, it was anticipated that 31.5% and 5% of patients could start Botox or neuromodulation, respectively. After Botox or neuromodulation failure, we assume discontinuation.

**Figure 2.** Treatment Progression

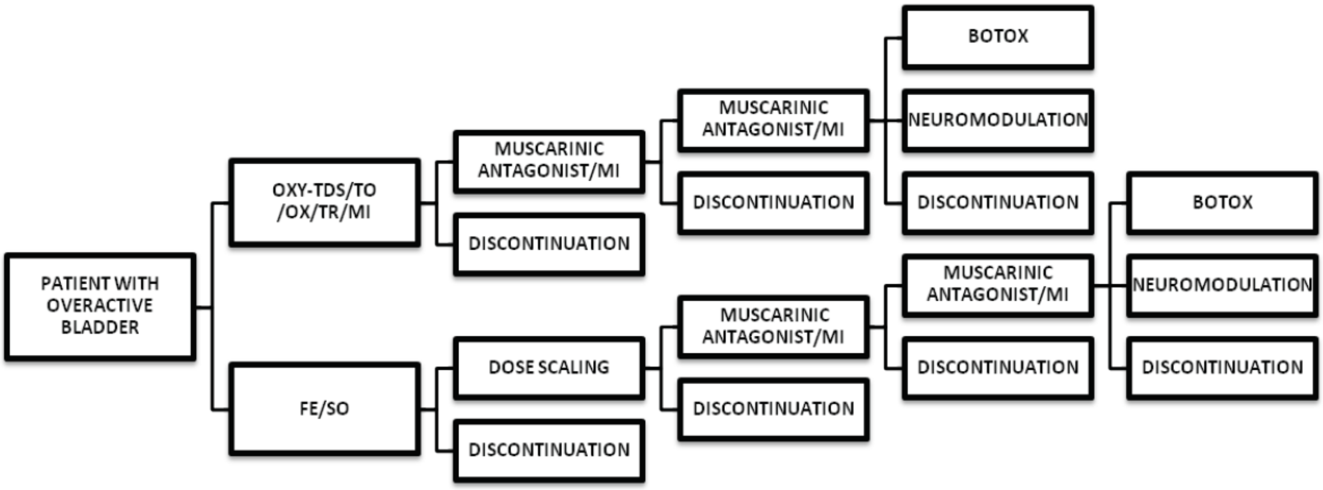

OXY-TDS: transdermal oxybutynin; TO: tolterodine; OX: oxybutynin; TR: trospium chloride; MI: mirabegron; FE: fesoterodine; SO: solifenacin

Each health state was assigned costs and clinical benefits (persistence, continence and QoL). Treatment effect was represented by patients' probability of moving from one state of health to another monthly.

## Probabilities

To estimate probabilities for the study, a literature search was conducted for Spanish and international efficacy studies, systematic reviews, observational studies and cost–effectiveness studies up to April 2017. To evaluate treatment persistence, an indirect meta-analysis of the most significant OAB studies was performed based on individual patient data simulated. Those studies that included persistence for at least one of the treatments evaluated in table or graph form were selected.[8,12,20-31] We did a multiple comparison treatment from real world evidence persistence studies. The results were weighted according to the sample size for each treatment, taking into account the time frame for each study. The DEALE method was applied to extrapolate persistence up to 5 years[32] (Table 1). As there was no viable way to distinguish between the persistence of the different presentations of SO (5 mg versus 10 mg) or FE (4 mg versus 8 mg), they were assumed to have the same persistence as that seen in the SO and FE studies, respectively. For all alternatives, it was assumed that persistent patients were continent and that patients who were going to discontinue early were incontinent the month before.

The efficacy of Botox and neuromodulation was based on a single study carried out in Spain that evaluated the cost–effectiveness ratio in a Spanish setting[33] and which clearly described the probabilities of therapeutic success of each alternative.

The probability of suffering from dry mouth or constipation with OXY-TDS came from its clinical trials[11,34] and the MATRIX phase 4 study.[35] By contrast, the probability of adverse effects with all other muscarinic antagonists came from a systematic review by Maman,13 while the probability of adverse effects with MI came from its phase 3 clinical trials.[36] It was assumed that 85% of patients who suffered from adverse effects ended up discontinuing treatment (Table 1).

**Table 1.** Parameters for the Model

| | | | | | | | | | |
|---|---|---|---|---|---|---|---|---|---|
| **PERSISTENCE** | | | | | | | | | |
| | **OXY-TDS** | **MI** | **TO** | **SO 5mg** | **SO 10mg** | **FE 4mg** | **FE 8mg** | **OX** | **TR** |
| Constant | 0.8707 | 0.6838 | 0.5989 | 0.6028 | | 0.6092 | | 0.4776 | 0.4615 |
| Lambda | –1.64E–03 | –1.49E–03 | –2.16E–03 | –1.97E–03 | | –1.70E–03 | | –2.32E–03 | –1.64E–03 |
| **ADVERSE EFFECTS (MONTHLY)** | | | | | | | | | |
| | **OXY-TDS** | **MI** | **TO** | **SO 5mg** | **SO 10mg** | **FE 4mg** | **FE 8mg** | **OX** | **TR** |
| Dry mouth | 1.8% | 2.8% | 10.9% | 10.8% | 22.9% | 11.6% | 22.3% | 29.6% | 12.3% |
| Constipation | 0.9% | 1.6% | 1.8% | 3.7% | 6.4% | 1.7% | 3.0% | 1.6% | 11.0% |
| **PROBABILITY OF A CHANGE IN TREATMENT DUE TO A LACK OF EFFICACY** | | | | | | | | | |
| | **OXY-TDS*** | **MI*** | **TO** | **SO 5mg** | **SO 10mg** | **FE 4 mg** | **FE 8mg** | **OX**** | **TR**** |
| 6 months | 29.6% | 29.6% | 29.6% | 18.3% | 18.3% | 22.1% | 22.1% | 23.8% | 23.8% |
| 12 months | 9.6% | 9.6% | 9.6% | 9.1% | 9.1% | 7.0% | 7.0% | 8.9% | 8.9% |
| 24 months | 7.8% | 7.8% | 6.3% | 5.8% | 5.8% | 6.3% | 6.3% | 4.9% | 7.3% |
| **PROBABILITY OF RESTARTING TREATMENT FOLLOWING DISCONTINUATION** | | | | | | | | | |
| | **OXY-TDS** | **MI†** | **TO** | **SO 5mg** | **SO 10mg** | **FE 4mg**** | **FE 8mg**** | **OX** | **TR** |
| 3 months | 9.3% | 9.3% | 8.1% | 8.4% | 8.4% | 8.0% | 8.0% | 7.9% | 9.8% |
| 6 months | 15.2% | 15.2% | 13.1% | 13.7% | 13.7% | 13.0% | 13.0% | 12.8% | 15.9% |
| 12 months | 17.2% | 17.2% | 14.9% | 15.5% | 15.5% | 14.8% | 14.8% | 14.6% | 18.1% |
| 18 months | 8.9% | 8.9% | 7.7% | 8.0% | 8.0% | 7.6% | 7.6% | 7.5% | 9.3% |
| 24 months | 5.7% | 5.7% | 4.9% | 5.1% | 5.1% | 4.9% | 4.9% | 4.8% | 5.9% |

*Assumed to be equal to TO; **Equivalent to overall value; †Assumed to be equal to OXY-TDS
OXY-TDS: transdermal oxybutynin; TO: tolterodine; OX: oxybutynin; TR: trospium chloride; MI: mirabegron; FE: fesoterodine; SO: Solifenacin

### Treatment Change and Restart Following Discontinuation

The probability of changing treatment due to a lack of efficacy came from a persistence study by Mauseth et al.[37] This study enabled us to calculate the probability of a change in treatment at different time cut-off points for up to a year of treatment. In addition, the estimation of the probability of long-term change was adapted based on the ratio seen after 24 months in a study by Chancelor et al.[38] OXY-TDS and MI were assumed to have the same probability as that seen in TO, except after 24 months, where MI was equated to the change value for OXY-TDS.[38] In all other cases where this probability could not be obtained (OX and TR), the overall value was assumed (Table 1).

Patients who discontinued could restart treatment. For this purpose, the probability of restarting following discontinuation was adapted from the Chancelor study3[8] for OXY-TDS, TO, SO, OX and TR. The same value was assumed for FE and SO, as separate results by presentation were not available. In addition, MI was assumed to be equal to OXY-TDS and FE was assumed to be equal to the overall value. It was assumed that 65% of patients who restarted did so with the same treatment that they discontinued.[38]

Once the patients who changed treatment had been identified, they were assigned prescription of other muscarinic antagonists or MI according to IMS sales data (Table 2).[39]

**Table 2.** Distribution of Treatment Prescribed Following Failure of a Treatment

| | FOLLOWING FAILURE OF: | | | | | | |
|---|---|---|---|---|---|---|---|
| **CHANGE TO TX:** | OXY-TDS | MI | TO | SO | FE | OX | TR |
| OXY-TDS | | 5.6% | 4.4% | 5.8% | 4.6% | 0.0% | 3.8% |
| MI | 33.2% | | 36.8% | 48.3% | 38.5% | 33.2% | 31.9% |
| TO | 13.8% | 19.5% | | 20.1% | 16.0% | 13.8% | 13.3% |
| SO 5 mg | 23.3% | 33.0% | 25.9% | | 27.1% | 23.3% | 22.4% |
| SO 10 mg | 11.9% | 16.8% | 13.2% | | 13.8% | 11.9% | 11.4% |
| FE 4 mg | 9.0% | 12.7% | 9.9% | 13.0% | | 9.0% | 8.6% |
| FE 8 mg | 8.8% | 12.5% | 9.8% | 12.8% | | 8.8% | 8.5% |
| OX | 0.0% | 0.0% | 0.0% | 0.0% | 0.0% | | 0.0% |
| TR | 0.0% | 0.0% | 0.0% | 0.0% | 0.0% | 0.0% | |
| | 100% | 100% | 100% | 100% | 100% | 100% | 100% |

OXY-TDS: transdermal oxybutynin; TO: tolterodine; OX: oxybutynin; TR: trospium chloride; MI: mirabegron; FE: fesoterodine; SO: Solifenacin

Quality of Life

To measure QoL loss deriving from OAB, each health state was assigned a different utility. Utility is a measure of patients' preferences in relation to their state of health where 1 equals perfect health and 0 equals death. QALYs are a measure that evaluates both survival and QoL (utility). This study defined utility as 0.823 for patients with therapeutic success (continent),[40] 0.737 for patients with therapeutic failure (incontinent) and 0.763 for patients who discontinued treatment.[41] A quality of life reduction of 0.037 points was applied in those patients who suffered from at least one adverse effect (dry mouth or constipation).[40]

**Use of Resources and Costs**

The healthcare resources evaluated were mainly limited to drugs, primary care or specialist visits and use of incontinence products, considering that they are directly associated with disease severity. In the model, it was assumed that continent patients required 0.17 incontinence products/day, incontinent patients required 1.864 incontinence products/day and patients who discontinued required 1.635 incontinence products/ day, based on a 2015 study by Nazir.[40] It was also assumed that patients in treatment had an average of three primary care and urology visits per year. When patients changed treatment, an additional visit to the urologist was assumed. The use of resources for patients with Botox or neuromodulation was adapted and updated from a Spanish OAB study.[33] The anticipated dose for Botox was always 100 international units.

All costs were expressed in euros for April 2017. Costs for previous years were updated using the consumer price index.[42] Pharmacological costs came from the Bot PLUS medicine database of the Spanish General Council of Official Colleges of Pharmacists (CGCOF) and were expressed in terms of manufacturer's suggested retail price (MSRP) incl. VAT based on Spanish recommendation42 (Table 3).

**Table 3.** Cost Parameters

| DRUG | MSRP incl. VAT | DAILY TREATMENT COST |
|---|---|---|
| OXY-TDS | €40.59 | €1.450 |
| MI | €45.12 | €1.504 |
| TO | €28.58 | €1.021 |
| SO 5 mg | €50.03 | €1.668 |
| SO 10 mg | €80.05 | €2.668 |
| FE 4 mg | €47.64 | €1.701 |
| FE 8 mg | €76.23 | €2.723 |
| OX | €4.15 | €0.2075 |
| TR | €10.71 | €0.357 |
| **BOTOX** | **VALUE** | **COMMENT** |
| Procedure | €1 102.43 | |
| Follow-up | €259.95 | Each 3 months |
| After failure | €271.31 | Monthly |
| **NEUROMODULATION** | **VALUE** | **COMMENT** |
| Procedure | €9 837.75 | Include device |
| Follow-up | €109.63 | Each 3 months |
| After failure | €223.88 | Monthly |
| **OTHER COSTS** | **VALUE** | **COMMENT** |
| General practitioner visits | €22.42 per visit | Average of 3 visits per year in persistent patients |
| Urologist visits | €56.68 per visit | Average of 3 visits per year in persistent patients |
| Incontinence pads | €0.58 per pad | |

MSRP: manufacturer's suggested retail price

### Sensitivity Analysis

To validate the robustness of the results, both an univariate and a probabilistic sensitivity analysis was performed for all the parameters (efficacy, discount probability, restarting probability, adverse events, use of resources, cost,…). The parameters evaluated in the scenario analysis were as follows:

- Utility for incontinent patients and patients who discontinued was 0.737.
- Presentations of SO were grouped and presented as an average between 5 mg and 10 mg.
- Distributions of changes in treatment were based on Quintiles-IMS prescription panel data.[42]

Probabilistic sensitivity analysis was additionally conducted in order to fully examine the combined effect of parameter uncertainty on the base-case result. For the probabilistic analysis, appropriate probability distributions following Briggs et al. (effectiveness and utility with a beta distribution and cost with a gamma distribution) were assigned to model parameters based on their respective means and standard errors.[42] Values for parameters were then sampled by Monte Carlo simulation with 1 000 iterations in each loop.

## RESULTS

The cumulative average time of initial treatment persistence was 16.7 months for OXY-TDS, 14.6 months for MI, 9.6 months for SO, 9 months for TO and FE, 8.9 months for TR and 6.1 months for OX. This meant that the cumulative percentage of discontinuation after 5 years was lower for OXY-TDS than for all other treatments, specifically, 63.1% for OXY-TDS, 66.67% for MI, 75.1% for TO, 76.2% for TR, 77.1% for SO, 77.2% for FE and 80.8% for OX. When QALYs were taken into account, the results were seen to range from 3.6740 to 3.6216, and OXY-TDS was the treatment with the most QALYs (Table 4).

**Table 4.** Cost-effectiveness Analysis

| | COST | | QALYs | | ICER |
|---|---|---|---|---|---|
| | OXY-TDS | COMPARATOR | OXY-TDS | COMPARATOR | |
| Mirabegron | €2,515.58 | €2,483.70 | 3.6740 | 3.6631 | €2,959.96 |
| Tolterodine | | €2,199.07 | | 3.6385 | €8,939.27 |
| Solifenacin | | €2,375.00 | | 3.6334 | €3,451.75 |
| Fesoterodine | | €2,473.55 | | 3.6332 | €1,313.96 |
| Oxybutynin | | €1,935.04 | | 3.6216 | €11,085.96 |
| Trospium chloride | | €1,958.87 | | 3.6345 | €14,101.87 |

ICER: Incremental cost–effectiveness ratio; OXY-TDS: Transdermal oxybutynin; QALY: quality-adjusted life year

At 5 years, the direct cost was €1 935.04 for OX, €1 958.87 for TR, €2 199.07 for TO, €2 375.00 for SO, €2 473.55 for FE, €2 483.70 for MI and €2 515.58 for OXY-TDS (Table 4). The higher cost for OXY-TDS was primarily due to its greater persistence and patient follow-up time. In addition, the cost distribution for each treatment was different (Figure 3). Except for OXY-TDS and MI, the cost of incontinence products represented more than 50% of the average total cost. The treatments with the greatest impact on the cost of prescription of a second or third first-line drug were TO, OX and TR.

**Figure 3.** Distribution of Total Cost after 5 Years

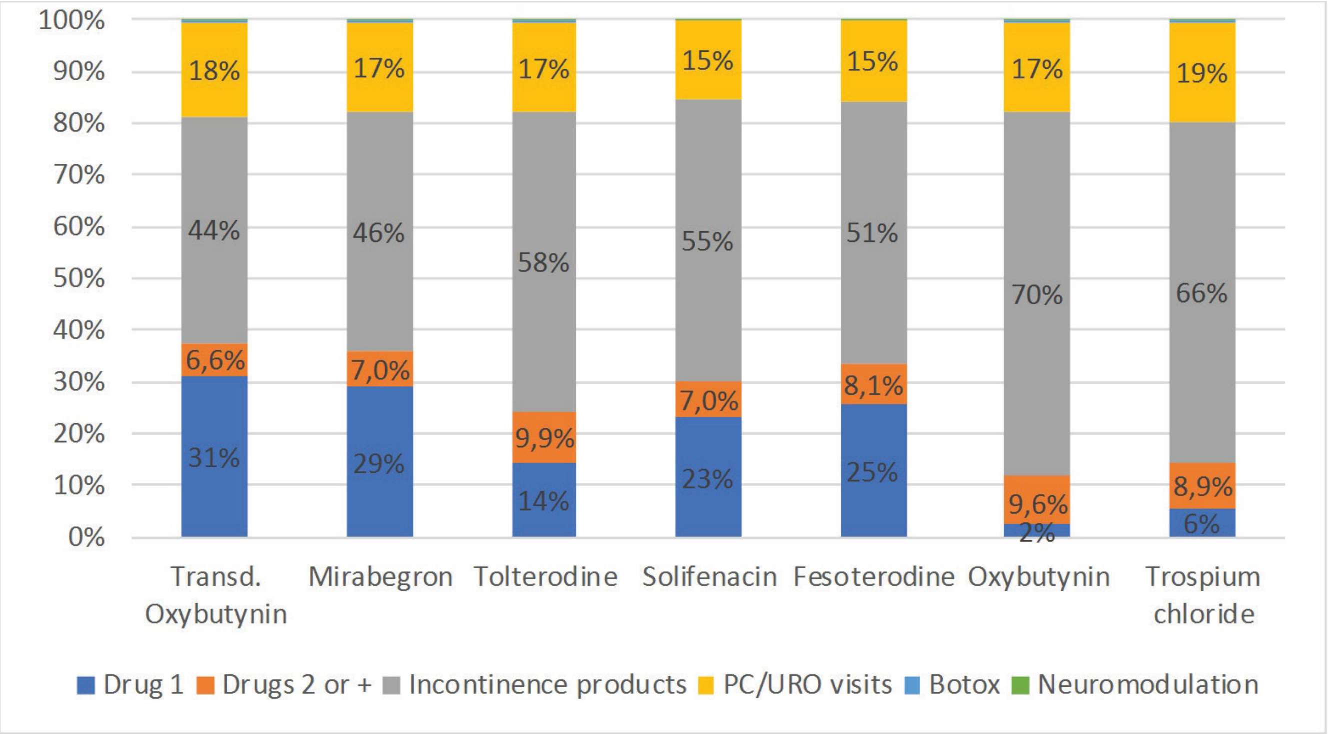

The ICER for OXY-TDS compared to FE was €1 313.96/QALY gained, compared to MI was €2 959.96/QALY gained, compared to SO was €3 451.75/QALY gained, compared to TO was €8 939.27/QALY gained, compared to OX was €11 085.96/QALY gained and compared to TR was €14 101.87/QALY gained (Table 4). Therefore, OXY-TDS was highly cost-effective as it showed a value well below the willingness to pay thresholds used in Spain (€25 000[16]-€30 000/QALY[17]).

The univariate and probabilistic sensitivity analysis confirmed the results for the base case in such a way that when utilities for incontinent patients and patients who discontinued were compared, the ICER ranged from €935/QALY gained to €10 104/QALY gained and was more favorable if it fit for OXY-TDS. In the case of grouping presentations of SO, the ICER was seen to decrease substantially to €1 812/QALY gained. When the distribution of changes in treatment was modified, OXY-TDS was seen to remain highly cost–effective, although the ICER underwent some modifications (FE €1 387.02/QALY gained, MI €3 010.26/QALY gained, SO €3 583.62/QALY gained, TO €12 240.91/QALY gained, OX €12 784.69/QALY gained and TR €14 096.33/QALY gained).

## Discussion

Although phase 3 clinical trials are standards for clinical evidence, it is increasingly necessary to be thoroughly familiar with the value of drugs, including under less-than-ideal circumstances. This has led to a proliferation of comparative effectiveness studies evaluating OAB treatment persistence, such as cost–effectiveness studies. This study, following that line of research, is the first cost–effectiveness study published in Spain comparing drugs recommended as first-line medications by the guidelines of the Spanish Association of Urology (AEU).[1] Another cost–effectiveness study in a European setting, based on a meta-analysis by Maman[13], was built, demonstrating that mirabegron was cost–effective for the United Kingdom's national health system.[40] Due to the existing differences between the British and Spanish national health systems in terms of costs and clinical practice aside, that study cannot be readily compared to our own, since it directly assumed a fictitious persistence for MI differing from that seen in persistence studies.[8,28-31] By contrast, the information for our study is the most complete and the closest to the reality seen in a Spanish healthcare setting for all treatments evaluated. One strength of the Nazir et al. study[40] is that it featured transition probabilities to break down the severity of episodes of incontinence and urinary frequency into 25 states of health, whereas for our study this was not possible.

## Limitations

Economic evaluation models are studies of secondary sources that, like systematic reviews, have a certain amount of validity, provided that the studies used for this purpose have that internal and external validity. Therefore, our study was not free of these limitations inherent to economic evaluations performed using pharmacoeconomic models.

Another limitation of our study was rooted in a dearth of more studies for OXY-TDS evaluating persistence with a larger sample size, or even studies from which the probability of transition between a gradient of states of health associated with frequency of urination and episodes of urination could be obtained. The reason why there is a limited number of studies with OXY-TDS is that its marketing is restricted to certain countries, which include Spain. Even so, the fact that we were able to refer to some persistence studies enabled us to more reliably approximate results in actual clinical practice.

These results may be validated in long-term persistence studies in a Spanish setting that gather actual information on the use of resources and costs. Thus, an estimate with real cost and effectiveness data could be prepared.

## CONCLUSIONS

OXY-TDS led to more QALYs gained, due to its persistence and tolerability, and was a highly cost–effective treatment, as it showed an ICER well below the thresholds assumed in Spain.[16,17]
